# Is Telemedicine our cup of tea? A nationwide cross-sectional survey regarding doctors’ experience and perceptions

**DOI:** 10.12669/pjms.37.5.3970

**Published:** 2021

**Authors:** Laima Alam, Mafaza Alam, Amina Mannan Malik, Varqa Faraid

**Affiliations:** 1 Laima Alam, FCPS. Consultant Gastroenterology, Bahria Town International Hospital, Rawalpindi, Pakistan; 2 Mafaza Alam Registrar Operative Dentistry, Armed Forces Institute of Dentistry, Rawalpindi, Pakistan; 3 Amina Mannan Malik Shifa International Hospital, Islamabad, Pakistan; 4 Varqa Faraid Shaheed Zulfiqar Ali Bhutto Medical University, School of Dentistry, Islamabad, Pakistan

**Keywords:** Chronic disease, Developing countries, Infrastructure, Survey, Telemedicine

## Abstract

**Objectives:**

To evaluate the experience and perceptions regarding Telemedicine and the perceived barriers among medical doctors.

**Methods:**

This cross-sectional survey was carried out by enrolling practicing doctors of Pakistan with experience of ≥6 months by sending a validated and piloted questionnaire through email. Data collection was done from 10^th^ October to 9th November 2020 after taking ethical approval from the concerned authorities. Data was analysed using SPSS v. 19.0.

**Results:**

Two-hundred-forty responses were received with a response rate of 63%. Female participants (62.8%) were in majority and most of the participants were working in urban (88.5%) or semi-urban (9%) locality in either teaching (35.9%) or tertiary care hospitals (34.6%). Seventy-three percent of the doctors didn’t receive formal training with more than half of the doctors reporting non-availability of infrastructure and specific hardware. A large number of the participants were concerned regarding the non-availability of regulatory bodies, evaluations and accreditations of the service providers, the risks of malpractice, missed-diagnosis, prescription errors and medico-legal issues. The availability of specific infrastructure was statistically related to the hospital setup, locality and the specialty of the participants. Lack of technological literacy and infrastructure were considered the main constraints for the public in using telemedicine.

**Conclusion:**

Evidence of effectiveness of telemedicine across different fields is inconsistent and lacks technical, legal, cultural and ethical considerations. Inadequate training, low level of technological literacy and lack of infrastructure are the main barriers in implementing tele-health. High-quality evidence based studies are required for practical and long-term policies.

## INTRODUCTION

Healthcare, being a rapidly evolving field, requires economical, efficacious with good reproducibility, practical and high-quality solutions for optimal patient care on individual as well as communal levels.^1^ Telemedicine, though not a recent entity in the medical field, has been hailed as a billion dollars industry with further potential to improve and expand. Telemedicine has aroused interest in the international markets due to its ability to overcome barriers in the way of assessing quality healthcare.^2^

Telemedicine includes services like tele-consultation, tele-monitoring, tele-counseling, tele-education, tele-care, tele-psychiatry and tele-rehabilitation that can serve clients remotely and widely.^1^ Distant healthcare can be used to realign chronic disease management for easy availability of quality care with less in-person hospital visits and cost-effective health modules.^3^

Pakistan, being a predominantly developing country, lags far behind in formulating and implementing sustainable healthcare policies.^4^ Despite being a concomitant commodity to conventional healthcare in developed countries, comparatively lesser efforts are done for similar utilities in the third world countries.^5^ Lesser return of investment (ROI), socio-economic barriers, poor adaptability and the unavailability of proper technological infrastructures are some of the local and international hurdles.^5^

Multiple research papers are available regarding the need for telemedicine and the “knowledge-attitude-practices” studies encompassing doctors but none have been formulated to assess the reason for reluctance towards implementing telemedicine in Pakistan. In this nationwide cross-sectional survey we tried to evaluate the ground realities responsible for poor acceptability of Telemedicine among doctors and the perceived barriers making this novel healthcare technology a failure.

## METHODS

This cross-sectional survey was carried out by enrolling practicing doctors working in Pakistan with clinical experience of ≥6 months through convenience sampling after acquiring ethical approval from the concerned department (905/Trg-ABP1K2 dated 1/10/2020). The participants were encouraged to share the survey through social-media for maximum participation. The survey was completed in one month i.e; from 10^th^ October to 9th November 2020. The questionnaire was developed by authors after relevant literature review.^4^-^8^ It was reviewed by two medical education experts for content validity. The survey was piloted among 10 doctors before putting it to test. The questionnaire was sent through email, a reminder was given to the participants after one week of no response and the candidates were dropped who failed to respond after another seven days.

The questionnaire consisted of three parts; demographics with availability of basic infrastructure for tele-health services, 17 questions evaluating the general perception and experience of telemedicine with a five-point Likert scale and the perceived barriers at public level.

The sample size was calculated with margin of error set at 5%, confidence level at 95% and an anticipated frequency (response distribution) of 50% using OpenEpi sample size calculator. To measure the internal consistency of the instrument, Cronbach’s alpha was calculated which produced a value of 0.86. Data was statistically described in terms of frequencies and percentages. Chi square test and Fisher exact test were used to compare qualitative data. All statistical analyses were performed using SPSS v 19.0. All p values ≤0.05 were considered statistically significant.

## RESULTS

**Fig.1:**
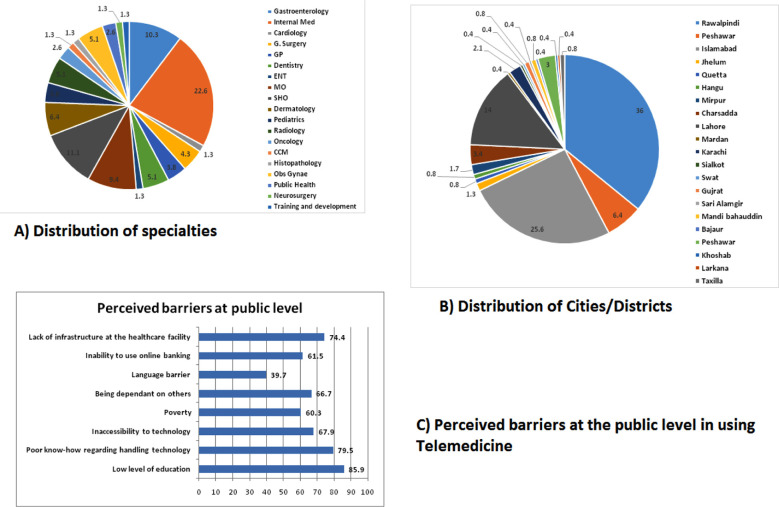
Distribution of participants according to location and medical specialty (A-B). Perceived barriers to the acceptability of telemedicine at public level (C).

A total of 380 questionnaires were sent, out of which 240 surveys were received back, making a response rate of 63%. Two-hundred-thirty four surveys were complete and the six incomplete responses were discarded. The distribution of specialties and cities/districts is shown in Fig.1. Female participants (62.8%) were in majority and most of the participants were working in urban (88.5%) or semi-urban (9%) locality in either teaching (35.9%) or tertiary care hospitals (34.6%). Seventy-three percent of the doctors didn’t receive formal telemedicine training with more than half of the doctors reporting non-availability of infrastructure and specific hardware (Table-I).

**Table-I:** Demographics and availability of basic infrastructure of telemedicine (n=234).

Variables	Frequency (%)	P value
***Gender***		
Male	87(37.2)	≤0.001
Female	147(62.8)	
***Age (years)***		
≤30	153(65.4)	≤0.001
31-40	66(28.2)	
41-50	12(5.1)	
>50	3(1.3)	
***Experience (years)***		≤0.001
<1	30(12.8)	
1-3	102(43.6)	
4-6	45(19.2)	
7-9	30(12.8)	
≥10	27(11.5)	
***Locality***		≤0.001
Rural	6(2.6)	
Urban	207(88.5)	
Semi-urban	21(9)	
***Work setup***		≤0.001
Private clinic	36(15.4)	
Primary healthcare	15(6.4)	
District hospital	18(7.7)	
Tertiary care hospital	81(34.6)	
Teaching hospital	84(35.9)	
***Telemedicine specific training***		≤0.001
Yes	48(20.5)	
No	171(73.1)	
Don’t know	15(6.4)	
***Availability of infrastructure***		≤0.001
Yes	75(32.1)	
No	123(52.6)	
Don’t know	36(15.4)	
***Availability of specific hardware***		≤0.001
Yes	63(26.9)	
No	150(64.1)	
Don’t know	21(9)	
***Availability of specific software***		≤0.001
Yes	138(59)	
No	63(26.9)	
Don’t know	33(14.1)	
Affordability for the setup		≤0.001
Yes	138(59)	
No	45(19.2)	
Don’t know	51(21.8)	
***Affordability for the patients***		≤0.001
Yes	120(51.3)	
No	57(24.4)	
Don’t know	57(24.4)	
***Means to measure effectiveness of telemedicine***		
Yes	84(35.9)	
No	51(21.8)	
Don’t know	99(42.3)	
***Need for physical examination***		≤0.001
Yes	213(91)	
No	21(9)	

A large number of the participants were concerned regarding the non-availability of regulatory bodies, evaluations and accreditations of the service providers, the risks of malpractice, missed-diagnosis, prescription errors and medico-legal issues (Table-II). The availability of specific infrastructure and necessary equipment was statistically related to the hospital setup, locality and the specialty of the participating doctors (Table-III) with smaller hospitals/clinics running at primary healthcare level being severely deficient in all facilities. The perceived affordability of tele-health services was also statistically related to the specialty of the participating doctors and their work set-up.

**Table-II:** Evaluation of telemedicine services using a five-point scale.

Survey questions	Strongly agree	Agree	Don’t know	Disagree	Strongly disagree	p
Do you believe telemedicine set up is expensive?	9(3.8)	27(11.5)	120(51.3)	45(19.2)	33(14.1)	≤0.001
Do you believe telemedicine has high cost of maintenance?	21(9)	39(16.7)	108(46.2)	45(19.2)	21(9)	≤0.001
Do you believe your society has a high resistance to change?	90(38.5)	69(29.5)	51(21.8)	12(5.1)	12(5.1)	≤0.001
Do you believe there is unavailability of required infrastructure for telemedicine?	63(26.9)	78(33.3)	60(25.6)	24(10.3)	9(3.8)	≤0.001
Do you believe there is lack of standard for comparison while using telemedicine?	81(34.6)	54(23.1)	72(30.8)	12(5.1)	15(6.4)	≤0.001
Do you believe there is lack of regulatory bodies for telemedicine?	87(37.2)	72(30.8)	48(20.5)	18(7.7)	9(3.8)	≤0.001
Do you believe there is lack of common care protocol for telemedicine?	48(20.5)	84(35.9)	72(30.8)	15(6.4)	15(6.4)	≤0.001
Do you believe there is lack of accreditation of service providers (evaluate, validate, certify)?	63(26.9)	57(24.4)	84(35.9)	21(9)	9(3.8)	≤0.001
Do you believe there is lack of regulations to avoid malpractice?	99(42.3)	63(26.9)	51(21.8)	6(2.6)	15(6.4)	≤0.001
Do you believe your practice of telemedicine would be covered by your indemnity?	21(9)	42(17.9)	75(32.1)	45(19.2)	51(21.8)	≤0.001
Do you believe there is lack of user friendly interface?	48(20.5)	78(33.3)	72(30.8)	24(10.3)	12(5.1)	≤0.001
Do you believe there would a risk to privacy and confidentiality?	42(17.9)	69(29.5)	78(33.3)	33(14.1)	12(5.1)	≤0.001
Are you concerned about missed diagnosis?	102(43.6)	84(35.9)	24(10.3)	15(6.4)	9(3.8)	≤0.001
Are you concerned about medico-legal issues?	102(43.6)	51(21.8)	42(17.9)	30(12.8)	9(3.8)	≤0.001
Are you concerned about prescription errors?	60(25.6)	87(37.2)	57(24.4)	24(10.3)	6(2.6)	≤0.001
Are you concerned about the lack of anthropometric measures and vitals in case patient video-calls from home?	90(38.5)	84(35.9)	33(14.1)	18(7.7)	9(3.8)	≤0.001
Are you concerned about the limited comfort with sensitive examination?	69(29.5)	90(38.5)	45(19.2)	18(7.7)	12(5.1)	≤0.001

**Table-III:** Relation of demographics with availability of basic infrastructure for telemedicine services.

Variables	Locality (p)	Nature of setup (p)	Specialty of the participating doctors (p)
Specific training	0.28	0.003	≤0.001
Availability of specific infrastructure	0.002	0.007	≤0.001
Availability of specific hardware	0.06	0.01	≤0.001
Availability of specific software	0.008	≤0.001	≤0.001
Affordability for the set-up	0.17	≤0.001	≤0.001
Affordability for the patients	0.03	0.002	≤0.001
Means to measure effectiveness of telemedicine services	0.03	0.002	≤0.001
Maintenance cost	0.001	≤0.001	≤0.001

Low level of public education, poor know-how regarding handling technology and lack of infrastructure were considered the main constraints for the public in using telemedicine (Fig.1).

## DISCUSSION

Tele-medicine is a novel technology for the poor third world countries and has a strong potential to bring about quintessential changes to healthcare facilities if used wisely.^9^ A systemic review by Bashshur R et al showed that tele-health services decreased the use of unnecessary antibiotics and re-admissions, increased the return visits at hospitals for necessary follow-up, increased smoking cessation rate and helped to reduce unnecessary referrals by 40%. Despite all these promising outcomes, mortality was unaffected and the availability of standardized communication facilities did not improve patient attendance.^10^

Many of the participants in our study were not aware of the financial implications and the running/maintenance costs involved in telemedicine, the phenomenon that was also studied in a local study^2^, proving that our medical community lacks familiarity with latest innovations. Despite the lack of this knowledge, many of the doctors in our study reported that telemedicine is an affordable service for both their set-ups and patients. In contrast, the doctors from specialized medical fields like Gynaecology and Obstetrics, Cardiology, Gastroenterology, Ophthalmology etc believed that tele-health might not be a cost-effective mode, probably because of the sophisticated technology required for evaluation and monitoring.

An emerging concern is the lack of robust studies regarding the cost analysis and its implications on the budgets of lower to middle income countries.^11^ Monitoring of chronic diseases has been historically considered cost-effective but the studies (and consequently the results) are sketchy.^12^ Setting up tele-health facility has been seen to be far more expensive than the running and maintenance cost.^7^ No consensus is available for the cost effectiveness of tele-health^7^ and vigorous studies are required for quality and control.

In our study only the junior doctors (who could be redeployed and were not a part of a specialized unit) and those in Public Health Department reported receiving specific training. The training was also found statistically related to the nature of set-up i.e, the doctors working in larger teaching and tertiary care hospitals reported availability of infrastructure and specific training. The rural and semi-urban set-ups have been studied to benefit more from tele-health,^13^ owing to logistics and funds allocation, but have been largely neglected in the poor counties just like other basic necessaties.^14^ Interestingly, the availability of specific hardware like electronic stethoscopes, ophthalmoscopes and high-quality digital cameras were deficient even in those set-ups that were practicing telemedicine on regular basis which explains the reluctance of specialized fields like Cardiology, Gastroenterology, Dentistry, Ophthalmology etc towards tele-consultations. The specialties including Dermatology, Radiology and preventive medicine did not need these gadgets and reported higher participation with availability of satisfactory framework.

It was interesting to see that many of the participants showed their lack of knowledge towards the basic facets of this novel healthcare facility including common care protocols, indemnity, standards for comparison, accreditation of service providers and risks to patient privacy. This illiteracy might be one of the biggest confounders responsible for the reluctance on part of the doctors and lack of prompting for conceivable policies at national level.^2^ An equally large number of doctors were concerned regarding the medico-legal implications associated with missed diagnosis, prescription errors, sensitive examinations and lack of anthropometric measures/vitals in our study which could be mitigated by effective triaging, training, redeployment of trained nurses and home monitoring.^7^,^8^

Acceptability towards an innovative technology has always been the main hurdle not only in the developing country but also the developed ones.^15^ Our study also reported a high resistance to change as perceived by the doctors but the availability of tele-health services in a particular set-up helped to mitigate the fear and confusion among public, making it more acceptable. The same phenomenon was also stated in a systemic review that urged for user friendly interface, incorporating local languages in to the applications and training of the personnel.^14^ This was contradictory to a study from Africa that showed a high acceptance towards tele-psychiatry consultations among women,^16^ making it necessary to conduct larger randomized studies with regional and cultural consideration.

The participating doctors reported lack of literacy and infrastructure as the most devastating elements responsible for poor acceptability of Tele-health at the public level. A similar study demonstrated the effect of poor infrastructure and resources as the main barrier in the development of Tele-medicine in the developing countries of Africa, Americas and the South-East Asia.^14^ This was in contrast to an indigenous study by Ahmed A et al that reported familiarity with technology was not an issue for the adoption of tele-health facilities.^2^

The practice of medicine and healing is intrinsically related to patient’s confidentiality, privacy and respect with sensitive examinations requiring utmost consideration and civility.^17^ Though, as many as 91% of the participants in our study claimed physical exam was necessary for their practice and diagnosis, an equal number were concern regarding the sensitivity of this issue.

Disregard to the local culture, poor training and evaluation systems and lack of continual assistance and guidance are all the major pitfalls common for developing countries.^14^ Telemedicine may not be suitable for all medical conditions and should be used in collaboration with the conventional healthcare facilities.^7^,^18^ Telemedicine has proven benefit in rapid triaging, tele-education, tele-rehabilitation, tele-psychiatry, monitoring of chronic diseases and tele-consultations among doctors for specialist opinion.^4^,^7^,^18^ Also careful patient triaging is needed for patient satisfaction and ultimately the success of a program.^19^

The greatest strength of this study is a good mix of all specialties with varied experiences, from different set ups and regions of the country as shown in Fig.1. Also rather than testing the basic knowledge of tele-medicine, doctors’ point of view regarding the perceived barriers was explored in detail. The limitation of the study is convenience sampling and participants were encouraged to share the survey through email for maximum participation.

## CONCLUSION

Evidence of effectiveness of telemedicine across different fields is inconsistent and lacks technical, legal, cultural and ethical considerations for the developing countries. Inadequate training, low level of technological literacy and lack of infrastructure are the main barriers in implementing tele-health. High quality evidence based studies are required for practical long-term policies.

### Author’s Contribution:

**LA:** Contributed to the idea, questionnaire, data collection, literature review, statistical analysis and drafting the manuscript.

**MA:** Contributed to data collection and questionnaire.

**AM, VF:** Contributed to data collection.

All the authors take equal responsibility for the accuracy and integrity of the work.
